# Periostitis

**Published:** 1854-04

**Authors:** D. B. Whipple


### *Periostitis.* By D. B. Whipple, M. D.

I have been induced to make a few observations and suggestions upon the treatment of this affection in the acute stage, and although the experience which induces the venture may be too brief to warrant them, I trust the observations incidentally made, will prove the means of eliciting further expression upon the subject.

My experience obliges me to confess that with all the remedial means I have been able to employ, the treatment of periostitis, in the acute stage, even under the most favorable circumstances, apparently, often proves unsuccessful. There seems to be an agent wanting within the pale of the dentist that will enable him to meet the indications of treatment more decidedly and promptly.

Admitting our aid is sought when a case presents in this stage, the object doubtless is to produce resolution, and I believe to produce this, our most effectual means is local depletion, and the leech is the agent prescribed. Now, it is upon this point that I wish to express myself particularly.

For illustration we will suppese a case: A patient applies for whom, a year previous, we treated the exposed pulp of a central incisor; the utmost care was exercised in every manipulation of the operation, and the case promised entire success, but now he complains of suffering extreme pain in it, and upon examination we find the periosteum inflamed, and in the inflammation in the acute stage, our diagnosis being made, the patient is directed to have leeches applied. Here we certainly lose control of the patient, at least he passes from our serveillance—we transfer him to another for aid. The inference seems to follow that the dentist does not possess all the remedial agents requisite to meet the demand made upon him, in the treatment of the teeth and their diseases. Surely there is not a dentist who has not wished it in his power to afford that relief, which he is obliged to seek from another in these cases. The indulgence of that desire induced me to experiments. Although my experience has been brief, my success induces me to annouuce it, trusting that the result of further experience will prove that it was not prematurely made.

Unquestionably the leech is the best means for the local abstraction of blood from the inflamed part about a tooth, but I think an artificial substitute can be employed to subserve the purpose quite as well. Something of suitable shape, upon the principle of the cupping glass,* with adequate exhausting power, would enable the operator to meet a case, as satisfactorily as with the natural leech. An article termed the dental leech introduced some years ago, is upon this principle, and with this instrument I have been enabled to treat several cases successfully. Many who have used them complain of their inefficiency, and I think it is attributable, altogether, to insufficient lancing. Slight incisions will not answer the purpose; the flow of blood obtained is slight, and owing to its fibrinous condition, coagulation is readily favored, and the exhausting power of the artificial leech is not sufficient to prevent it, in fact, it only serves to add to the existing irritation of the part.

My plan, and upon which is based all my success in their use, is to lance very deeply and freely, and for the purpose, I use the physicians' spring lancet, setting it to cut entirely through the integument, and making in some instances a cru-

* The writer is aware that instruments similar to the one he suggests have been introduced.

cial incision. My preference for using such a lancet is, that I can make the required incisions more direct, and so quickly that very little, if any, pain is occasioned. By thus severing a great number of the congested blood-vessels, a large flow is established, and favoring the determination and relaxation, by bathing the part with warm water, I am enabled to obtain as much as we would require by the natural leech. I will admit that it is a troublesome operation, but not more so than many others that present themselves to us, and I think the satisfaction of being able to afford the relief ourselves, and of being spared the necessity of transferring to another that which legitimately belongs to our especiality, is sufficient compensation of itself.

It may be asked, are there no other means of meeting such a case? Surely there are; but I believe it is conceded that local depletion is the most prompt and effective. Inflammation in this locality is the same in its characteristics, as the acute form of pleuritis, pneumonia, etc., and will admit of similar sytematic treatment. All the antiphlogistic remedies may be resorted to; the counter irritants and discutients also —but from the confined and circumscribed character of the inflammation in periostitis, local depletion will meet the case more decidedly. After the application of the leech, I direct the part to be bathed with

| | | |
|---|---|---|
| ℞. | Sulph. Ether, - - - - - | ℥ ss |
| | Gum Camph., - - - - | ʒ ss |

By the ether we have the anodyne influence, and refrigerant also, from its volatile properties, and a tonic action is exerted upon the dilated and relaxed vessels. The camphor is added for its discutient properties. (This combination may be used as a prophylatic remedy, when slight vascular excitement is evinced.)

My purpose by this paper, is chiefly to describe my mode of using the lancet in conjunction with the artificial leech, in the acute stage of periostitis, and the adjunctive treatment I refer to, merely from its incidental connection. To treat of periostitis, in its different stages, would involve a reference to all the established principles of surgery. It is in the acute stage that our services are generally solicited, and if we are not able to meet the demand here, true inflammation ensues, and then a chronic condition is established, which we cannot treat with any positive promise of success.

